# 罕见*COX7A2L-ALK*融合突变晚期肺腺癌1例病例报告并文献复习

**DOI:** 10.3779/j.issn.1009-3419.2023.102.15

**Published:** 2023-04-20

**Authors:** YUAN Jiao, PAN Ruili, ZHONG Wei, WANG Mengzhao

**Affiliations:** ^1^610213 成都，成都市第七人民医院呼吸内科（袁娇）; ^1^Department of Respiratory Disease, Chengdu Seventh People’s Hospital, Chengdu 610213, China; ^2^100730 北京，中国医学科学院，北京协和医学院，北京协和医院呼吸与危重症医学科（袁娇，潘瑞丽，钟巍，王孟昭）; ^2^Department of Pulmonary and Critic Care Medicine, Peking Union Medical College Hospital, Peking Union Medical College and Chinses Academy of Medical Science, Beijing 100730, China

**Keywords:** 罕见ALK融合突变, ALK抑制剂, 肺肿瘤, COX7A2L-ALK融合突变, Rare ALK fusion mutation, ALK inhibitors, Lung neoplasms, COX7A2L-ALK fusion mutation

## Abstract

肺癌是全世界发病率和死亡率最高的肿瘤，随着下一代测序（next generation sequencing, NGS）检测技术的发展，越来越多的罕见间变性淋巴瘤激酶（anaplastic lymphoma kinase, ALK）融合突变患者被检测出来。本文报道了北京协和医院收治的1例罕见COX7A2L-ALK（C2:A20）融合突变的肺腺癌晚期患者，同时检索2014年1月1日-2021年3月31日发表的罕见ALK融合突变的病例报道，探讨ALK抑制剂对罕见ALK融合突变患者的疗效。本例患者一线给予口服塞瑞替尼后病情好转，疗效评价为部分缓解（partial response, PR）。通过上述检索方法检索出符合文献19篇，共报道22例罕见ALK融合突变，结合本例，对23例进行汇总分析。分析结果显示，ALK抑制剂对罕见ALK融合突变的客观有效率为82.6%（19/23），疾病控制率为95.7%（22/23）。罕见ALK融合突变晚期肺腺癌患者可以从ALK抑制剂治疗中受益。

## Abstract (en)

Lung cancer is the most common in incidence and mortality worldwide. With the development of next generation sequencing (NGS) detection technology, more and more patients with rare anaplastic lymphoma kinase (ALK) fusion mutations were detected. A case of advanced lung adenocarcinoma with rare COX7A2L-ALK (C2:A20) fusion detected by NGS was reported in Peking Union Medical College Hospital, and all cases with rare ALK fusion mutations were searched from medical datebase from January 1, 2014 to March 31, 2021, to investigate the treatment of rare ALK fusion mutations with ALK inhibitors. The best response of the patient was assessed as partial response (PR) with Ceritinib treatment. By literature review, 22 cases of rare ALK fusion were reported in 19 articles. Combined with this case, 23 cases were analyzed. The objective response rate (ORR) was 82.6% (19/23) and disease control rate (DCR) was 95.7% (22/23) for rare ALK fusions patients treated with ALK inhibitors. Lung adenocarcinoma patients with rare ALK fusion could benefit from ALK inhibitors.

间变性淋巴瘤激酶（anaplastic lymphoma kinase, ALK）是一种酪氨酸激酶，3%-7%的非小细胞肺癌（non-small cell lung cancer, NSCLC）中存在ALK基因位点的融合突变^[1]^。Soda等^[2]^于2007年首次报道了棘皮动物微管相关蛋白样4（echinoderm microtubule associated protein like 4, EML4）-ALK融合突变，之后又发现了KIF5B-ALK、KLC1-ALK、TFG-ALK、ALK-PTPN3等突变^[3⇓⇓⇓⇓-8]^。随着测序技术的发展，ALK融合的新伴侣基因也越来越多报道^[9]^。罕见ALK融合患者使用ALK抑制剂疗效不确定，本文报道了1例罕见的COX7A2L-ALK融合突变晚期肺腺癌，并进行了文献复习。

## 1 病例资料

患者女，48岁，因咳嗽、左侧胸痛1个月就诊。体检发现双侧锁骨上肿大淋巴结。无吸烟史。胸部计算机断层扫描（computed tomography, CT）（2021-01-08）示左肺门占位，多发纵隔淋巴结肿大，肺内散在小结节，左侧胸膜增厚。骨扫描（2021-02-01）示左侧第9后肋局限性放射性摄取增高灶，考虑骨转移灶。头增强磁共振成像（magnetic resonance imaging, MRI）（2021-01-29）未见明显异常。气管镜检查（2021-01-15）示左固有上叶黏膜充血粗糙，开口外压性狭窄。于左固有段黏膜活检及支气管内超声引导针吸活检术（endobronchial ultrasound-guided transbronchial needle aspiration, EBUS-TBNA）。黏膜活检病理为慢性炎症；TBNA淋巴结活检病理为肺腺癌，免疫组化（immunohistochemistry, IHC）：Ventana ALK-D5F3（+），CK7（+），P40（-），甲状腺转录因子1（thyriod transcription factor 1, TTF-1）（+），未行荧光原位杂交（fluorescence in situ hybridization, FISH）。诊断为左肺腺癌（cT_3_N_3_M1_b_, IVB）ALK（+）。东部肿瘤协作组（Eastern Cooperative Oncology Group, ECOG）体能评分为1分。下一代测序（next generation sequencing, NGS）（Illumina nextseq 500测序系统覆盖68基因）检测结果提示：COX7A2L-ALK（C2:A20）融合突变，丰度11.61%；同时有腺瘤性息肉病（adenomatons polyposis coli, APC）基因外显子16错义突变NM-000038.5:c.2258A>G（p.H753R），丰度56.26%；RET内含子区域突变NM-020975.4:c.1263+7C>G，丰度39.71%；FLT3内含子区域突变NM-004119.2:c.1206-9T>A，丰度46.49%；TP53外显子6错义突变NM-0000546.5:c.623A>T（p.D208V），丰度6.31%。

治疗上予以塞瑞替尼450 mg每日一次随餐口服作为一线治疗。患者口服塞瑞替尼1个月后复诊，诉咳嗽和左侧胸痛症状明显缓解，复查胸部CT（2021-03-04）示左肺内占位、纵隔淋巴结明显缩小，肺内小结节和左侧胸膜增厚消失。根据实体瘤疗效评价标准（Response Evaluation Criteria in Solid Tumor, RECIST）1.1版疗效评价为部分缓解（partial response, PR），见图1。门诊随访至2022年10月，患者一般状况良好，无疾病进展（progressive disease, PD），无进展生存期（progression-free survival, PFS）超过了20个月。

**图 1:**
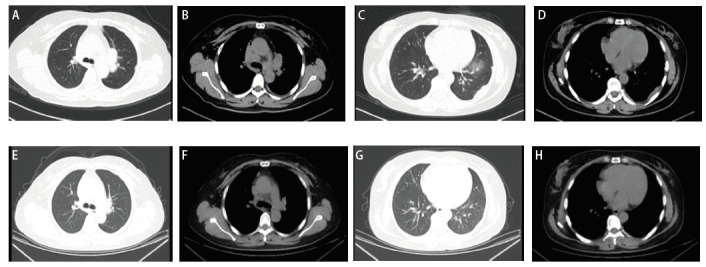
患者治疗前后胸部CT的改变。 A、B：塞瑞替尼治疗前左肺门占位和纵隔淋巴结肿大；C、D：塞瑞替尼治疗前肺内结节和胸膜增厚；E、F：塞瑞替尼治疗1个月后左肺占位和纵隔淋巴结明显缩小；G、H：塞瑞替尼治疗1个月后肺内小结节和左侧胸膜增厚消失。

## 2 讨论

以“novel ALK rearrangement”“Lung cancer”和“ALK inhibitor”为关键词在PubMed数据库检索，以“罕见ALK融合”和“肺癌”在中国期刊网全文数据库（CNKI）、万方医学进行文献检索。检索时间为2014年1月1日-2021年3月31日，同时排除了以下情况：（1）NSCLC以外的肿瘤；（2）病例报道不详细；（3）治疗过程中未使用ALK抑制剂治疗。通过上述检索方法共检索出符合文献19篇，共报道22例罕见ALK融合突变，结合本例，对23例进行汇总分析。均为3’端ALK激酶结构的罕见融合伴侣或罕见双ALK融合。22例病例的临床资料见表1^[10⇓⇓⇓⇓⇓⇓⇓⇓⇓⇓⇓⇓⇓⇓⇓⇓⇓-28]^。结合本例共23例患者分析，男性15例（65.2%），女性8例（34.8%）。患者平均年龄（52.7±11.7）岁，中位年龄54岁。有吸烟史7例，不吸烟9例，是否吸烟不详7例。所有患者都是肺腺癌，使用ALK抑制剂时均为IV期。患者临床表现有咳嗽咳痰、呼吸困难、疼痛、体重减轻等，影像表现为肺部占位、胸水等，较一般肺癌患者无特殊。ALK融合突变检测方式上均采用NGS检测方法，ALK融合突变类型中有3例为STRN-ALK类型，2例为BCL11A-ALK类型（1例出现在双融合突变患者中），其他类型的突变均为1例。其中3例为ALK双融合突变，分别为EML4-ALK和BCL11A-ALK、EML4-ALK和BIRC6-ALK、DYSF-ALK和ITGAV-ALK。同时接受ALK FISH检测12例，其中5例阳性和7例阴性。同时接受ALK IHC检测8例，其中7例阳性和1例阴性。有关治疗和转归，23例患者ALK抑制剂作为一线治疗18例，二线治疗4例，三线治疗1例。一线治疗选择使用的ALK抑制剂中克唑替尼占78.3%（18/23），阿来替尼占17.4%（4/23），塞瑞替尼占4.3%（1/23）。ALK抑制剂的客观疗效评价为：客观有效率（objective response rate, ORR）为82.6%（19/23），疾病稳定（stable disease, SD）率为13.0%（3/23），疾病控制率为95.7%（22/23），PD率为4.3%（1/23）。客观疗效为PD的患者的突变类型为CMTR1-ALK（C2;A20）。有2例患者在首个ALK抑制剂进展后使用了其他ALK抑制剂，仍显示出一定的疗效。PD患者的中位随访时间为18个月，仅有5例患者报道了PD时间或死亡，因此无法计算PFS和总生存期（overal survival, OS）。

**表1:** 罕见ALK融合患者的临床特征和ALK-TKI的客观疗效

Reference	Type of ALK fusion	ALK IHC	ALK FISH	n	Gender	Age (yr)	Smoking status	Patholgical type	Stage	ALK-TKI	Treatment lines	ORR	PFS (mon)	Progressed or death
Shan et al.^[10]^	BIRC6-ALK	+	-	1	Male	45	Yes	AD	IV	Crizotinib	1	PR	8	NA
Drilon et al.^[11]^	SOCS5-ALK	NA	-	1	Male	61	NA	AD	IV	Crizotinib	1	SD	NA	Yes
	CLIP4-ALK	NA	-	1	Male	29	NA	AD	IV	Crizotinib	1	PR	5	No
Ali et al.^[12]^	EIF2AK-ALK	-	-	1	Female	71	No	AD	IV	Crizotinib	1	PR	28	No
	PRKAR1A-ALK	+	+	1	Male	67	Yes	AD	IV	Crizotinib	1	PR	7	No
	PPMAB-ALK	NA	NA	1	Female	NA	NA	AD	IV	Crizotinib	1	PR	NA	NA
Jiang et al.^[13]^	GCC2-ALK (G13:A20)	NA	NA	1	Female	28	No	AD	IV	Crizotinib	1	PR	18	Yes
										Ceritinib	2	SD	8	Yes
										Alectinib	3	SD	6	Yes
										Lorlatinib	4	SD	4	NA
Feng et al.^[14]^	TNIP2-ALK	NA	NA	1	Female	49	No	AD	IV	Crizotinib	1	PR	2	No
Fei et al.^[15]^	CENPA-ALK	+	+	1	Female	60	NA	AD	IV	Crizotinib	1	PR	2	No
Tian et al.^[16]^	BCL11A-ALK	NA	NA	1	Female	64	No	AD	IV	Crizotinib	1	PR	6	No
Chen et al.^[17]^	SRBD1-ALK (S6, A20)	+	+	1	Male	59	Yes	AD	IV	Crizotinib	1	PR	10	No
Chen et al.^[18]^	SOS1-ALK (S2, A20)	NA	NA	1	Male	52	No	AD	IV	Crizotinib	1	PR	6	No
Zhang et al.^[19]^	CUX1-ALK (S6, A20)	NA	NA	1	Male	66	NA	AD	IV	Crizotinib	2	PR	20	NA
Zhu et al.^[20]^	VKORC1L1-ALK	NA	-	1	Male	54	Yes	AD	IV	Crizotinib	2	PR	60	Yes
										Alectinib	3	PR	5.3	NA
Yin et al.^[21]^	DYSF-ALK; ITGAV-ALK	NA	+	1	Male	44	Yes	AD	IV	Crizotinib	2	SD	3	No
Du et al.^[22]^	CMTR1-ALK (C2; A20)	NA	-	1	Male	75	Yes	AD	IV	Crizotinib	2	PD	1	Yes
Hu et al.^[23]^	VIT-ALK	+	+	1	Female	64	NA	AD	IV	Alectinib	1	PR	5	No
Nakanishi et al.^[24] ^	STRN-ALK	+	-	1	Male	51	No	AD	IV	Alectinib	1	SD	NA	Yes
Yang et al.^[25]^	STRN-ALK	NA	NA	1	Male	59	No	AD	IV	Crizotinib	3	PR	8	No
Su et al.^[26]^	STRN-ALK	NA	NA	1	Male	64	No	AD	IV	Alectinib	1	PR	19	NA
Zhong et al.^[27]^	EML4-ALK; BIRC6-ALK	NA	NA	1	Male	60	NA	AD	IV	Alectinib	1	PR	2	No
Qin et al.^[28] ^	EML4-ALK; BCL11A-ALK	NA	NA	1	Male	29	Yes	AD	IV	Crizotinib	1	PR	13	NA

ALK: anaplastic lymphoma kinase; TKI: tyrosine kinase inhibitor; IHC: immunohistochemistry; FISH: fluorescence in situ hybridization; ORR: objective response rate; PR: partial remission; SD: stable disease; PD: progressive disease; PFS: progression-free survival; AD: adenocarcinoma; NA: not available; EML4: echinoderm microtubule associated protein like 4.

ALK基因重排是重要的NSCLC的驱动基因，本文报道了1例COX7A2L-ALK融合患者，之前未见报道。同时通过文献复习，总结了23例罕见ALK融合患者的情况。COX7A2L基因编码COX7A2L蛋白，也称为SCAFI（SC-specific assembly factor I）、COX7RP，负责与线粒体呼吸链（mitochondrial respiratory chain, MRC）复合体动态结合，以使MRC功能适应代谢变化^[29]^。MRC功能障碍在肿瘤发生中起重要作用。在肝癌研究^[30]^中发现COX7RP过度表达，诱导细胞周期进程和上皮间质转化（epithelial-mesenchymal transition, EMT）并抑制细胞凋亡而促进肝细胞癌的生长和转移，并预示了肝癌患者的预后不良。COX7A2L-ALK融合很可能是肺癌发生发展的关键驱动因素。

23例患者均通过NGS诊断，提示了NGS对融合基因罕见伴侣的检出具有优势。其他方法包括ALK IHC和ALK FISH，本文中ALK IHC检测8例，其中87.5%（7/8）阳性，ALK FISH检测12例，其中41.7%（5/12）阳性，提示了免疫组化在发现罕见ALK重排中优于FISH。Shan等^[10]^报道了1例应用FISH检测阴性但ALK Ventana-D5F3 IHC检测ALK阳性的患者，运用NGS检测出罕见BIRC6-ALK融合突变。Drilon等^[11]^报道FISH检测阴性的2例肺腺癌患者中，再次运用NGS检测分别发现为SOCS5-ALK和HIP1-ALK融合。在NSCLC患者中ALK融合的准确检测至关重要，IHC检测方法仍是最经济简便的首推检测方法，而FISH不足以识别所有的ALK融合突变病例，NGS在ALK基因融合具有很多优势，但目前，关于NGS检测样本的质控等一系列问题我国还没有明确的规范，且费用高，NGS技术包括全基因组测序、全外显子组测序、热点基因变异的NGS小/大panel检测等，可以根据实际情况是否使用NGS来筛查ALK相关基因的融合突变情况。DNA-based和RNA-based NGS临床意义不同，在DNA-based NGS未能检出的融合基因，可能在RNA-base NGS中检测到。因为有些融合基因仅发生在RNA层面，或基因在DNA层面融合丰度低，或是存在长内含子或重复序列的重合，这时RNA-based NGS可以有效避免这些融合基因的漏检。精准治疗的关键在于精准检出可治疗靶点的变异，研究^[31]^表明一些驱动基因融合的融合伴侣会影响治疗应答和疗效，而RNA测序可以确定融合伴侣和融合外显子。若能将DNA-based和RNA-based NGS结合使用，可提高临床获益率。在斯隆-凯特琳纪念肿瘤中心的一项研究^[32]^中，232例DNA-based NGS组织活检驱动突变阴性的NSCLC患者中，通过RNA-based NGS方法检测发现仍有15.5%（36/232）的患者检出可进行靶向治疗的融合基因，且经过靶向治疗后，临床获益率达80%。

目前批准的ALK抑制剂包括克唑替尼、塞瑞替尼、阿来替尼、布加替尼、恩沙替尼和劳拉替尼等。对罕见ALK融合类型，首个ALK抑制剂的ORR高达82.6%，并不差于常见ALK融合类型。首个ALK抑制剂更多患者选择了克唑替尼，可能与克唑替尼是第一个ALK抑制剂有关。Jiang等^[13]^报道中罕见GCC2-ALK（G13:A20）融合突变的患者予以克唑替尼一线治疗PR 18个月，PD给予二线化疗，三线予以塞瑞替尼治疗3个月后，患者因不能耐受改为阿来替尼治疗，PD予以四线劳拉替尼治疗。

Du等^[22]^报道罕见CMTR1-ALK融合患者对ALK抑制剂克唑替尼无效，研究者提出可能是由于CMTR1-ALK融合不能翻译成导致癌症发生的驱动蛋白，表明ALK融合类型和功能的重要性。Nakanishi等^[24]^的报道中携带STRN-ALK罕见融合突变的患者使用一线阿来替尼治疗，最佳疗效为SD，换用化疗后患者死亡。此患者STRN-ALK融合，但同时存在Vimentin的高表达，研究者提出有可能提示组织转化例如表皮-间质转化可能是效果不佳的原因。Zhu等^[20]^报道在术后复发携带VKORC1L1-ALK罕见突变患者二线予以克唑替尼治疗达5年，在出现ALK T1151K耐药突变后予以阿来替尼治疗再次有效。

我们报道了1例罕见COX7A2L-ALK融合突变NSCLC，考虑患者有临床意义的基因改变为COX7A2L-ALK融合突变，在ASCEND-8临床研究中，塞瑞替尼450 mg随餐给药方式下疗效得到了大幅度提升，总体缓解率达到78%^[33]^。塞瑞替尼于2018年获得国家药品监督管理局（National Medical Products Administration, NMPA）批准上市，2020年5月NMPA批准塞瑞替尼进入ALK阳性局部晚期或转移性NSCLC患者的一线治疗。结合成本效用分析我们选择塞瑞替尼作为此患者的一线治疗。本病例一线使用塞瑞替尼治疗后疗效达到PR，目前已随访20个月，仍处于随访中。ALK抑制剂对罕见融合类型的疗效尚无大规模的临床数据，值得临床医生去关注。随着检测技术的发展，如何选择NGS、IHC和FISH以及如何评价各种方法的结果，都应该仔细评估。选择哪个ALK抑制剂也应进一步探讨。本文提示罕见ALK融合突变晚期肺腺癌患者可以从ALK抑制剂治疗中受益。
